# Strong Association of Perceived Chronic Stress with Leadership Quality, Work–Privacy Conflict and Quantitative Work Demands: Results of the IMPROVE*job* Study

**DOI:** 10.3390/bs15050624

**Published:** 2025-05-03

**Authors:** Julian Göbel, Lukas Degen, Karen Minder, Monika A. Rieger, Birgitta M. Weltermann

**Affiliations:** 1 Institute of General Practice and Family Medicine, Medical Faculty of the University of Bonn, Venusberg-Campus 1, 53127 Bonn, Germany; julian.goebel@ukbonn.de (J.G.);; 2 Bonn Network Health Services Research, 53127 Bonn, Germany; 3 Institute of Occupational and Social Medicine and Health Services Research, University Hospital Tuebingen, Wilhelmstr. 27, 72074 Tuebingen, Germany; monika.rieger@med.uni-tuebingen.de

**Keywords:** general practitioner, practice personnel, work demands, work–privacy conflict, leadership

## Abstract

The health of primary care professionals is crucial for the health of populations. A lower number of general practitioners per 1000 patients correlates with higher patient mortality. Challenging work demands, work–privacy conflict, and poor leadership quality are associated with higher perceived chronic stress and/or burnout in physician populations. However, studies investigating the influence of all three factors in a single quantitative model are lacking. This study analysed the associations between the mentioned parameters and perceived chronic stress among general practice personnel based on baseline data of the cluster-randomized IMPROVE*job* study. It comprised 60 German general practices with 366 participants (84 general practice leaders, 28 employed physicians, 254 practice assistants). Perceived chronic stress (TICS-SSCS), leadership quality (LMX-7, FIF), work–privacy conflict (COPSOQ), and quantitative and emotional work demands (COPSOQ) were measured with validated questionnaires. The factors associated with lower perceived chronic stress were identified using a multilevel regression model approach. The model showed a significant association with less work–privacy conflict (*p* < 0.001, *β* = 0.31), lower quantitative work demands (*p* < 0.001, *β* = 0.28), and good leadership quality (*p* < 0.001, *β* = −0.22). Especially transformational leadership with the dimension ‘innovation of the leader’ was associated with lower perceived chronic stress. The data support the importance of high-quality leadership as a protective factor for perceived chronic stress among general practice personnel, which needs to be considered in future leadership interventions in this setting.

## 1. Background

The well-being of the primary care workforce is fundamental to sustainable health care systems (Søvold et al., 2021) and the health of populations (Starfield et al., 2005). However, this is at risk in many nations (Dewa et al., 2017) such as the UK and Germany, which are suffering from a shortage of primary care physicians (Federal Association for Statutory Health Insurance Physicians, 2023; Iacobucci, 2019; Owen et al., 2019). A comprehensive investigation of general practices in England found a significant negative correlation between patient mortality and the number of general practitioners (GP) per 1000 patients (−4.31, 95% CI −6.8 to −1.8) (Baker et al., 2016). In addition, a retrospective observational study in England showed an association between an increased turnover in general practice and lower patient satisfaction with the practice (−1.3; 95% CI −1.6 to −1.1), higher emergency attendances per 100 patients (1.8; 95% CI 1.5 to 2.1), and lower availability of same-day appointments (−10.6; 95% CI −11.4 to −9.0) (Parisi et al., 2023). Key factors for the GP demand–supply mismatch are early (Rabatin et al., 2016) and regular retirement of GPs (Nusbaum, 2009), demographic changes with longevity of patients, more elderly people requiring care (S. A. Cohen, 2009), and increased GP turnover rates due to psychological strains such as burnout and perceived chronic stress (CS) (Willard-Grace et al., 2019). To address these issues, many countries have initiated policies of various kinds, such as the NHS Long Term Plan in the United Kingdom (NHS, 2019) or regulations of the German Social Code Book V (SGB V §75a) (Kassenärztliche Bundesvereinigung, 2017).

The magnitude of the problem is illustrated by a large number of studies, which address several outcomes of psychological distress among GPs, such as burnout and perceived chronic stress (Dreher et al., 2019; Viehmann et al., 2017). Internationally, most studies have focused on the outcome burnout which is defined as a result of chronic stress at the workplace according to the International Statistical Classification of Diseases and Related Health Problems (ICD-11) (Graen & Uhl-Bien, 1995). A recent meta-analysis of 17 studies among 4497 French GPs showed a prevalence of 48% for burnout and 5% for severe burnout (Kansoun et al., 2019). About 50% of 683 Irish GPs experienced burnout symptoms (O’Dea et al., 2017). In a European-wide study in 12 countries by the European General Practice Research Network, 12% of 1393 family doctors scored high on all dimensions of burnout (Soler et al., 2008).

As defined in the ICD-11, the precursor of burnout is perceived chronic stress in the workplace. However, this is poorly studied in GP populations. In one of the few studies available, the prevalence of high perceived chronic stress was 25% in a German study of 214 general practitioners, which was much higher than in the German general population (11%) (Viehmann et al., 2017).

Psychological distress at work is mainly caused by three factors, namely hindrance work demands (Kim et al., 2023), work–privacy conflict (WPC), and poor leadership quality, as described in recent reviews by Meredith including 141 studies and Patel (Meredith et al., 2022; Patel et al., 2018). Regarding work demands, a German study of general practice teams with 214 GPs and 550 practice assistants identified long working hours as a cause of perceived chronic stress (Viehmann et al., 2017). In a qualitative study with 24 Canadian family physicians, practice management challenges were identified as stressors (Lee et al., 2009). Similarly, administrative tasks contributed to higher perceived stress levels in a recent cross-sectional study of 2037 GPs in Switzerland with higher perceived stress levels in younger (<40 years of age) compared to older GPs (Glättli et al., 2021).

In addition to such high work demands, work–privacy conflicts, also known as work–life conflict or work–family conflict are relevant (Hämmig et al., 2009; Netemeyer et al., 1996). WPC highlights potential conflicts between professional and personal lives, for example work-related stressors, such as overtime, negatively affecting family and private life (Garthus-Niegel et al., 2016). As published prior, GP leaders reported more than 50% higher work–privacy conflict scores than the German reference population with over 200,000 participants (Göbel et al., 2022; Lincke et al., 2021). These difficulties in balancing professional and private life are a predictor for a high degree of psychological distress, e.g., among 1755 Swiss (OR of 2.2) (Goehring et al., 2005) and 422 US primary care physicians (Rabatin et al., 2016).

In addition to quantitative work demands and work–privacy conflict, leadership quality is a major driver for perceived chronic stress and burnout. From a scientific perspective, leadership aspects are conceptualized in various models. The well-known Full Range of Leadership Model encompasses the dimensions transactional, transformational, and negative leadership (Bass, 1999). In the medical context, high transformational leadership is associated with a lower incidence of adverse patient outcomes (Boamah et al., 2018). Another theory, the Leader–Member Exchange Theory, focuses on the relationship between leaders and team members (Graen & Uhl-Bien, 1995). Higher-quality leader–member exchange was associated with lower levels of emotional exhaustion in a sample of 343 German healthcare employees (Gregersen et al., 2016). A recent systematic review of 15 studies showed associations between better leadership and lower levels of burnout in US medical professionals (Meredith et al., 2022). For example, a study of 762 resident physicians from the Mayo Clinic reported that a 1-point increase in leadership quality was associated with a 9% decrease in the odds of experiencing burnout (Dyrbye et al., 2020). Similar results were obtained in a large meta-analysis of 22 studies with 6861 participants from various occupational fields: higher leadership quality was strongly associated with lower perceived leader and subordinate stress levels (overall correlation of r_s_ = −0.35) (Harms et al., 2017).

So far, studies of GPs psychological distress focus either on work demands, work–privacy conflict, or leadership quality, but studies addressing all three aspects in one single quantitative model are lacking. This study draws on baseline data from the cluster-randomised controlled IMPROVE*job* study with more than 360 professionals from 60 primary care practices (Degen et al., 2021). It investigates the role of work demands, work–privacy conflict, and leadership quality as predictors for perceived chronic stress in general practice personnel.

## 2. Methods

This cross-sectional study analysed baseline data from our IMPROVE*job* study which comprised 366 professionals (84 practice leaders, 28 employed physicians, and 254 practice assistants) from 60 practices in the North-Rhine region of Germany. The IMPROVE*job* study was a participatory, cluster-randomised, controlled intervention trial which aimed at improving job satisfaction of German GP practice teams as primary outcome. In addition, secondary outcomes such as leadership quality, work demands, chronic stress, and work–privacy conflict were measured. The IMPROVE*job* intervention comprised multimodal, innovative leadership training on leadership skills, communication, and workflows (Weltermann et al., 2020). The baseline data collection took place before randomisation and was completed in January 2020 prior to the COVID-19 pandemic. Workshops for intervention practices were completed in August 2020. The study protocol and baseline results are published (Degen et al., 2021; Weltermann et al., 2020).

### 2.1. Outcome Measures

Questionnaires requested the following data:

- Sociodemographic and work characteristics: sex (m/f), age (in years), leadership responsibility (yes/no), part-time vs. full-time work.
- Perceived chronic stress: The validated TICS-SSCS (Trier Inventory for Chronic Stress—Screening Scale for Chronic Stress) measures the perceived burden of chronic stress in the last three months. It consists of 12 items with a 5-point Likert scale answering format (e.g., ‘In the last three months, how often did you experience fear of not being able to perform your duties?’; 0 = ‘never’ to 4 = ‘very often’) and is suitable for use in work-related diagnostics for both employees and self-employed persons (Schulz et al., 2004). A sum score of all 12 items is calculated, resulting in a score from 0 to 48 with 0 meaning ‘never stressed’ and 48 meaning ‘very often stressed’. The internal validity of the TICS-SSCS is excellent, with a Cronbach’s alpha of 0.91 (Schulz et al., 2004).
- Work demands and work–privacy conflict: These were measured using the corresponding scale of the Copenhagen Psychosocial Questionnaire (German COPSOQ, version 2018) (Burr et al., 2019). The COPSOQ is a validated instrument for measuring psychosocial factors at work (Lincke et al., 2021). The quantitative demands scale consists of five items (e.g., ‘How often do you not have time to complete your work tasks?’) and has a high internal validity (Cronbach’s alpha = 0.81). The emotional demands scale is based on two items (e.g., ‘Do you have to deal with other people’s personal problems as part of your work?’) and has a high internal consistency (Cronbach’s alpha = 0.74). The work–privacy conflict scale consists of two items (e.g., ‘The demands of my work interfere with my home and family life.’) and has a high internal validity (Cronbach’s alpha = 0.92). The response options for the scales are: always, often, sometimes, seldom, never/hardly ever. Following the COPSOQ manual, these were transformed into a numerical scale from 0–100, with high values indicating strong quantitative demands, emotional demands, and work–privacy conflicts.
- Quality of Leadership: This was assessed using the LMX and FIF questionnaire.
- Leader–Member Exchange: The leader–member exchange questionnaire (LMX-7) measures the quality of relationships between practice leaders and their staff with seven items on a 5-point Likert scale (e.g., ‘How would you characterise your working relationship with your leader/your member?’). The LMX-7 reflects the widespread concepts of transactional and transformational leadership and is based on the Leader–Member Exchange Theory (Graen & Uhl-Bien, 1995). The scale is analysed by calculating a sum score of all seven items with results ranging from 7 to 35 (Schyns & Knoll, 2014). The resulting five score categories describe the quality of leader–member exchange: 7 to 14 = very low, 15 to 19 = low, 20 to 24 = moderate, 25 to 29 = high, 30 to 35 = very high (Northouse, 2021). The internal consistency is high (Cronbach’s alpha = 0.92) (Schyns & Knoll, 2014). Incidentally, question seven was missing for all employed physicians (n = 28), who were therefore excluded from LMX analyses.
- Integrative Leadership Questionnaire (FIF): The validated FIF questionnaire measured transformational and transactional leadership with 40 items answered on a 5-point Likert scale (e.g., ‘My manager communicates the meaning and background of upcoming tasks and goals.’). All scales are analysed by calculating a mean score, ranging from 1–5. Transformational and transactional leadership are reported as global mean scores and in sub-dimensions: innovation, team spirit, performance development, individuality focus, providing a vision and being a role model (transformational leadership), and goal setting and management by exception (transactional leadership) (Rowold & Poethke, 2017). For regression analysis, an overall score was created by averaging the practice leader’s self-assessment and the employees’ external assessment of their leader.

### 2.2. Statistical Analysis

To enable comparability of results, means and standard deviations of the scales are reported. Due to the clustered structure of the data (general practices), we calculated multi-level regression models with random intercepts using perceived chronic stress measured by TICS-SSCS as primary outcome.

The multi-level model is based on the following equation:

Yᵢⱼ = γ₀₀ + ∑ₖ γₖ₀·Xₖᵢⱼ + u₀ⱼ + ∑ₖ uₖⱼ·Xₖᵢⱼ + rᵢⱼ

with Yᵢⱼ: outcome variable for physician i in practice j and Xₖᵢⱼ: predictor k for physician i in practice j (k = 1, …, 7).

We conducted a stepwise approach with 3 models for a profound understanding of associations. Model 1 analysed the impact of quantitative and emotional work demands, work–privacy conflict and leadership on perceived chronic stress. Model 2 was a regression model which addressed associations between leadership styles and perceived chronic stress. Model 3 focused on subdimensions of leadership associated with perceived chronic stress of GPs and practice assistants. Models 2 and 3 were applied to the total population and to the subpopulation of practice assistants due to their high perceived chronic stress (Degen et al., 2021). All significant predictors were included in the linear regression models. This approach aimed at identifying the leadership dimensions that protect against high perceived chronic stress. Effect sizes were reported in R^2^ according to Cohen (J. Cohen, 1988).

To ensure comparability with other studies, we also calculated multiple OLS regression models given a low ICC (>0.05).

This study was first approved by the Ethics Committee of the Medical Faculty of the University of Bonn (reference number: 057/19, date of approval: 20 February 2019).

## 3. Results

### 3.1. Descriptive Analysis of Sociodemographic Parameters

Of the 60 participating practices, 21 were solo practices and 39 were group practices. At the practice level, the proportion of the participating staff ranged from 20.0 to 100% (mean = 73.4%). The mean age of the population was 44.4 years (*SD* = 12.8); practice leaders were 10 years older on average than practice staff and more likely to work full-time (see Table 1).

### 3.2. Work Demands, Work–Privacy Conflict, and Leadership (Model 1)

The overall mean perceived chronic stress score of the population was 19.02 (*SD* = 8.80, *median* = 19) (Degen et al., 2021). Practice assistants reported the highest perceived chronic stress. Quantitative demands were highest for practice leaders, followed by practice assistants and employed physicians. Practice leaders reported the highest emotional demands and the highest work–privacy conflict (Göbel et al., 2022). Also, practice leaders assessed their own leadership quality higher than it was rated by practice assistants (see Table 2 for details) (Schmidt et al., 2023).

A multilevel regression model respecting for the clustered data structure showed significant associations of better leadership (higher LMX score), (*p* < 0.001), lower quantitative demands (*p* < 0.001), lower work–privacy conflict (*p* < 0.001), higher age (*p* < 0.001), and having leadership responsibility (*p* < 0.01) with lower perceived chronic stress (Table 2).

To allow for comparison with other studies reporting coefficients of determination, we calculated a multiple OLS regression. This was possible because of a low ICC (>0.05). This analysis showed an adjusted R^2^ = 0.37. Single linear regression models of the significant predictors of perceived chronic stress yielded adjusted determination coefficients of R^2^ = 0.13 for leadership (*p* < 0.001), R^2^ = 0.21 for WPC (*p* < 0.001) and R^2^ = 0.23 for quantitative demands (*p* < 0.001). A multilevel analysis with the same variables only on the subpopulation of GP leaders showed similar results, with quantitative demands, leadership, and WPC being the variables most strongly associated with chronic stress. The additional multiple OLS regression showed an adjusted R^2^ = 0.45 (see Table 3 for details).

### 3.3. Associations of Leadership Dimensions with Perceived Chronic Stress (Models 2 and 3)

The regression model 2 shows that higher transformational leadership (FIF) (*b* = −2.68, *p* = 0.012), higher leader–member exchange (LMX-7) (*b* = −0.37, *p* = 0.024), and higher age (*b* = −0.10, *p* = 0.024) are significantly associated with lower perceived chronic stress (see Table 4).

The transformational leadership dimension was the most influential variable associated with perceived chronic stress (Model 2). Considering the influence of the subscales of transformational leadership, our multilevel model 3 showed a significant association of the subscale ‘innovation of the leader’ with CS, identifying a high grade of innovation as a protective factor for CS (*b* = −2.23, *p* = 0.018).

Because practice assistants report the highest levels of perceived chronic stress descriptively, we analysed this subgroup in more detail. In line with the results of models 2 and 3, transformational leadership (*b* = −3.56, *p* < 0.001) and especially innovation of the leader (*b* = −2.23, *p* = 0.018) were significantly associated with perceived chronic stress (regression models controlled for age and working full-time/part-time).

## 4. Discussion

Our analysis showed that low work–privacy conflict, low quantitative work demands, and high leadership quality were the strongest predictors for a low level of perceived chronic stress in a multilevel model resulting in a high adjusted R^2^ of 0.37. Our analysis was based on the idea that multi-parameter scenarios like GP practices need to be investigated with multi-parameter approaches. This is in line with a conceptual model by Linzer et al. showing various predictors of perceived stress among primary care physicians in 2009, including organisational and leadership factors (Linzer et al., 2009), and with a narrative review by Patel (Patel et al., 2018). However, neither of these publications reported any effect sizes of the identified predictors. The review by Patel et al. conceptualised three key aspects with sub-dimensions which are relevant for physician burnout: (a) organisational factors (according to the authors, e.g., quality of leadership), (b) personal characteristics (according to the authors, e.g., work–privacy conflict), and (c) work factors (e.g., quantitative demands) (Patel et al., 2018).

The importance of leadership in the explanation of perceived chronic stress or burnout is supported by various studies. In a large US Mayo Clinic study by Shanafelt et al., 2813 physicians were asked to rate their direct physician/scientific leader. The leadership ratings explained 11% of the variance in individual physician burnout and 47% of the variance in organisational satisfaction (Shanafelt et al., 2015). These are notable effects given that lower job satisfaction is strongly negatively correlated with chronic stress (Degen et al., 2021). These results on leadership are similar to our analysis, showing an explained variance of 13% of chronic stress as a necessary precursor of burnout in linear regression models. These are remarkably high coefficients compared to a Hungarian study of 350 general practitioners, which identified age, gender, and fewer years in practice as predictors of burnout. The regression models on different burnout dimensions showed an adjusted R^2^ of 0.023–0.031 (Adam et al., 2018). Likewise, a 2010 Norwegian longitudinal study examined predictors of physician burnout among 683 participants in a multiple regression model. Individual factors, work characteristics, and work–home conflict were also analysed, but leadership parameters were not taken into account (Langballe et al., 2011). Our study addressed leadership quality in combination with work demands and work–privacy conflict and helps to understand the development and prevention of perceived chronic stress in populations that have been shown to be highly burdened (Degen et al., 2021; Hapke et al., 2013) Interestingly, practice assistants reported higher chronic stress compared to practice leaders, despite their management responsibilities. This finding is consistent with Karasek’s well-known *job demand–control model*, which postulates that high job demands combined with high perceived control (job decision latitude) define an active job and promote the development of new behaviour patterns (physicians in our sample), whereas high job demands combined with low control promote the risk of psychological strain (practice assistants) (Karasek, 1979).

Regarding personal characteristics (work–privacy conflict, assigned according to Patel et al., 2018) and work factors (quantitative work demands), our results are in line with a Swiss study of 1755 primary care physicians, which used a logistic regression approach to identify work-related stressors together with job and psychosocial characteristics as potential sources of burnout (Goehring et al., 2005). The regression model in that study explained 19% of the variance. In comparison, our comprehensive multilevel model explained almost twice as much of the variance. This may be due to the inclusion of leadership predictors, which show a high regression coefficient in our model, supporting the importance of leadership in the context of general practice. Further analyses revealed that transformational leadership, including especially the ‘innovation of the leader’ sub-dimension, was the strongest protective leadership factor for perceived chronic stress. These results were confirmed for the particularly stressed subgroup of practice assistants. In our model, work–privacy conflict had a significant impact on the perceived chronic stress among GPs and practice assistants. Our bivariate correlation analyses suggest that the relationship between work–privacy conflict and perceived chronic stress is moderated by leadership.

Several work factors are related to perceived chronic stress in general practice including long working hours/high workload, pressure on practice teams, high levels of bureaucracy, poor workflows, emotional demands, and poor team culture (Kersting et al., 2019; Riley et al., 2021). Our analysis included these factors using the COPSOQ scales for quantitative and emotional demands. The comprehensive regression model showed that quantitative demands are a highly significant predictor, in line with the studies mentioned above (Kersting et al., 2019; Riley et al., 2021). In contrast, emotional demands were not significantly associated with perceived chronic stress in our analysis.

In 2017, Shanafelt and Noseworthy published nine organisational strategies to reduce physician burnout (Shanafelt & Noseworthy, 2017). These are mostly top-down and include both leadership and work–privacy balance aspects. However, the difficulty lies in transferring such frameworks addressing larger organisations to the context of general practice, as most GP practices in Germany, for example, are owner-managed. Interventions must therefore shift the focus from the organisation to the practice leader, and should consider teaching and training different leadership styles (transformational with a high degree of leader’s innovation), which have been shown to be particularly protective factors against perceived chronic stress.

### 4.1. Strengths and Limitations

We used a multilevel regression approach with several models to examine the dynamic work environment of general practices in terms of perceived chronic stress and its protective factors. Based on large data from 60 German general practices, the interplay of important parameters such as leadership was analysed, but transfer of results to other settings needs to be handled with caution. Our sample from the German North-Rhine region included both urban and rural areas. The socio-demographic characteristics of our GP leader population are similar to the national level (age: our population: 54.3% vs. national sample: 55.3%; female gender: our population: 52.4% vs. national level: 52.2%) (Kassenärztliche Bundesvereinigung, 2023). Nevertheless, the results are somewhat limited in their generalisability because there are regions in Germany with a lower density of GPs, which face additional organisational challenges. In addition, results are based on cross-sectional data, which do not allow for a causal interpretation.

### 4.2. Conclusion and Practical Implications

In future longitudinal studies and interventions, quantitative work demands, WPC and leadership need to be understood in more detail to develop targeted interventions. These interventions should aim at factors influencing the quality of the relationships between practice leaders and their teams including specific leadership styles and dimensions.

## Figures and Tables

**Table 1:** Sociodemographic characteristics (Degen et al., 2021) and workplace perceptions of the total population and stratified by occupational groups.

Sociodemographic Parameters	Total Sample	Practice Leader	Employed Physician	Practice Assistant
Variable	*n* = 366	*n* = 84	*n* = 28	*n* = 254
Female, %	87.1	52.4	78.6	99.6
Age in years, mean (SD)	44.4 (12.8)	54.3 (6.2)	44.8 (9.8)	41.0 (13.0)
Working full-time, %	52.0	90.5	28.6	41.5
Workplace perceptions				
Perceived chronic stress (TICS), $$$mean (SD) (primary outcome)	19.02 (8.80)	18.15 (8.13)	16.38 (7.60)	19.60 (9.10)
Quantitative demands (COPSOQ), mean (SD)	60.53 (16.92)	67.28 (15.22)	55.80 (18.54)	58.72 (16.70)
Emotional demands (COPSOQ), $$$mean (SD)	69.10 (21.25)	86.01 (11.64)	75.93 (14.80)	62.59 (20.97)
Work–privacy conflict (COPSOQ), mean (SD)	40.85 (31.51)	64.03 (29.96)	45.54 (30.28)	32.67 (28.35)
Leadership quality (LMX-7), $$$mean (SD)	26.72 (4.40)	28.10 (2.6)	n/a	26.70 (4.8)

**Table 2:** Multilevel regression model with effect of independent variables of leadership quality, quantitative demands, emotional demands, work–privacy conflict, age, leadership responsibility, and working full-time/part-time on dependent variable of perceived chronic stress (n = 366).

	*b*	*SE_b_*	*β*	*t*
Quantitative demands ***	**0.15**	**0.03**	**0.28**	**5.57**
Emotional demands	0.04	0.02	0.09	1.74
Work–privacy conflict ***	**0.09**	**0.02**	**0.31**	**5.64**
Leader–Member Exchange (LMX-7) ***	**−0.45**	**0.09**	**−0.22**	**−4.44**
Age ***	**−0.14**	**0.04**	**−0.20**	**−3.43**
Working as a practice assistant ** ^a^	**3.00**	**1.22**	**0.14**	**2.92**
Working full-time/part-time ^b^	1.12	0.90	0.06	0.45

Annotation: *** *p* < 0.001, ** *p* < 0.01; *b* = regression coefficient b; *SE_B_ =* standard error; *β* = standardised regression coefficient; *t* = t-value; ^a^ coded as: 1 = no, 2 = yes; ^b^ coded as 0 = full-time, 1 = part-time; significant variables printed in bold.

**Table 3:** Multilevel regression model with effect of independent variables of leadership quality, quantitative demands, emotional demands, work–privacy conflict, age, and working full-time/part-time on dependent variable of perceived chronic stress for GP leaders only (n = 84).

	*b*	*SE_b_*	*β*	*t*
Quantitative demands ***	**0.22**	**0.06**	**0.40**	**3.86**
Emotional demands	0.10	0.06	0.14	1.60
Work–privacy conflict *	**0.06**	**0.03**	**0.21**	**2.02**
Leader–Member Exchange (LMX−7) ***	**−1.02**	**0.28**	**−0.32**	**−3.65**
Age	−0.18	0.17	−0.13	−1.53
Working full-time/part-time ^a^	1.13	2.56	0.04	0.44

Annotation: *** *p* < 0.001, * *p* < 0.05; *b* = regression coefficient b; *SE_B_ =* standard error; *β* = standardised regression coefficient; *t* = t-value; ^a^ coded as 0 = full-time, 1 = part-time; significant variables printed in bold.

**Table 4:** Total study population (n = 366). Multilevel regression models on leadership dimensions (Model 2) and leadership subdimensions (Model 3) associated perceived chronic stress. Models were controlled for age, working full-time/part-time, and role in practice (practice assistant/physician).

**Leadership dimensions of associated with perceived chronic stress (n = 366) (Model 2)**				
	*b*	*SE_b_*	*β*	*t*
Transactional Leadership	0.12	0.80	0.01	0.15
Transformational Leadership *	**−2.68**	**1.06**	**−0.24**	**−2.53**
Leader–Member Exchange *	**−0.37**	**1.64**	**−0.18**	**−2.27**
Age *	**−0.10**	**0.04**	**−0.15**	**−2.28**
Working as a practice assistant ^a^	−0.47	1.40	−0.03	−0.34
Working full-time/part-time ^b^	−1.34	1.06	−0.08	−1.26
**Subdimensions of transformational leadership associated with perceived chronic stress (n = 366) (Model 3)**				
	*b*	*SE_b_*	*β*	*t*
Age	−0.09	0.05	−0.13	−1.94
Working full-time/part-time ^a^	−1.44	1.21	−0.08	−1.20
Innovation *	**−2.23**	**0.94**	**−0.22**	**−2.38**
Team spirit	−0.21	0.82	−0.03	−0.26
Performance development	0.35	0.76	0.04	0.46
Individuality focus	−0.89	0.77	−0.10	−1.16
Providing a vision	−0.83	0.94	−0.10	−0.88
Being a role model	0.03	0.90	0.00	0.03

Annotation: * *p* < 0.05; ^a^ coded as 1 = no, 2 = yes; ^b^ coded as 0 = full-time, 1 = part-time; significant variables printed in bold.

## Data Availability

There are no plans to grant access to full protocol, participant-level datasets, or statistical codes, as data contain potentially identifying information.
